# Blood Glucose Monitoring Biosensor Based on Multiband Split-Ring Resonator Monopole Antenna

**DOI:** 10.3390/bios15040250

**Published:** 2025-04-15

**Authors:** Dalia N. Elsheakh, EL-Hawary Mohamed, Angie R. Eldamak

**Affiliations:** 1 Electrical Department, Faculty of Engineering and Technology, Badr University in Cairo, Badr 11829, Egypthawarymohamed1010@gmail.com (E.-H.M.); 2 Microstrip Department, Electronics Research Institute (ERI), El Nozha 11843, Egypt; 3 Electronics and Communications Engineering Department, Faculty of Engineering, Ain Shams University, Cairo 11517, Egypt; angie.eldamak@eng.asu.edu.eg

**Keywords:** glucose levels, monopole antenna, non-invasive, multiband and sensor, continuous monitoring, split-ring resonator (SRR)

## Abstract

This paper introduces a novel-shaped, compact, multiband monopole antenna sensor incorporating an irregular curved split-ring resonator (SRR) design for non-invasive, continuous monitoring of human blood glucose levels (BGL). The sensor operates at multiple resonance frequencies: 0.94, 1.5, 3, 4.6, and 6.3 GHz, achieving coefficient reflection impedance bandwidths ≤ −10 dB of 4%, 1%, 3.5%, 65%, and 50%, respectively. Additionally, novel shapes of two SRR metamaterial cells create notches at 1.7 GHz and 4.4 GHz. The antenna is fabricated on an economical FR4 substrate with compact dimensions of 35 × 50 × 1.6 mm^3^. The sensor’s performance is evaluated using 3D electromagnetic software, incorporating a human finger phantom model and applying the Cole–Cole model to mimic the blood layer’s sensitivity to blood glucose variations. The phantom model is positioned at different angles relative to the biosensor to detect frequency shifts corresponding to different glucose levels. Experimental validation involves placing a real human finger around the sensor to measure resonant frequency, magnitude, and phase changes. The fabricated sensor demonstrates a superior sensitivity of 24 MHz/mg/dL effectiveness compared to existing methods. This emphasizes its potential for practical, non-invasive glucose monitoring applications.

## 1. Introduction

The main characteristic of diabetes is that it is typically brought on by extremely high or low blood glucose levels for an extended length of time [1,2]. Millions of people worldwide suffer from diabetes, a chronic illness. Over 60% of the global diabetes population lives in a “risk zone”, accounting for 463 million cases of the disease, according to the World Health Organization (WHO) and the International Diabetes Federation (IDF) [2]. To increase the accuracy of these findings, it is advised that this identification and monitoring can be done frequently. Finger prick tests and glucose monitoring are two methods that can be used to identify diabetics [3,4], albeit they are seen as intrusive and harmful to the patient. Blood glucose levels can rise or fall as a result of diabetes, which is characterized by poor glucose utilization. It is well known to be a long-term metabolic disease that impacts important body organs. Diabetes can cause potentially dangerous side effects, such as peripheral neuropathy, heart disease, renal failure, and visual loss if it is not well monitored [5]. An individual in good health should have blood glucose levels that fall within the normal range of 70–100 mg/dL during fasting and 70–140 mg/dL after meals. Hyperglycemia, or elevated blood sugar, and hypoglycemia, or decreased blood sugar, are both harmful. Hypoglycemia can cause weakness or even unconsciousness. Conversely, blood hyperglycemia may potentially be an emergency or result in diabetic complications [6]. Diabetes is a chronic illness that arises from either insufficient insulin production or inefficient insulin utilization by the body. As a result, regular blood glucose testing is essential in managing diabetes. Since diabetes cannot be permanently cured, the only treatments are lifestyle modifications and a nutritious diabetic diet [7].

The patient has discomfort from frequent blood glucose monitoring based on this unpleasant intrusive procedure, and invasive methods like laboratory and one-touch glucometers raise the risk of blood-related infections. Furthermore, diabetics have to pay for recordings and endure the monotony of doing the same tests repeatedly [8,9]. The description of conventional glucometers that need finger pricking has prompted research on non-invasive or minimally invasive methods. The accuracy and user discomfort of the current options, such as wrist monitors and forearm glucometers, are criticized. This raises the prospect of using microwave glucose testing methods for a genuinely non-invasive method. For non-invasive glucose monitoring, several technologies have been proposed, some of which have even advanced to the commercialization stage. These technologies include optical [10], transdermal techniques [11], thermal spectrum measurement [12], breath acetone analysis [13], impedance spectroscopy [13], graphene-based nano-sensors [14], electrochemical methods that measure the glucose level in urine [15,16,17], contact lenses [18], acoustic spectroscopy [19], Raman spectroscopy [20], or a combination of these techniques [21]. However, a drawback of several of these commercial technologies is that they are not entirely non-invasive; contact lenses and implantable devices are two examples, while a few others are launched as semi-invasive devices. They are also costly and place limitations on the user [22].

Because electromagnetic (EM) microwaves can pass through bodily tissues, they are also used in continuous non-invasive blood glucose measurement and management [23]. These kinds of systems primarily depend on variations in the permittivity (ε) of real and imaginary parts of tissues or target cells because variations in glucose concentration cause variations in the EM parameters of ISF beneath the skin, tears, sweat, blood in the arteries, and so forth [24]. Additionally, these devices enable users to check their blood sugar levels without having to prick their fingertips every day [25]. There are several approaches to detecting electromagnetic properties based on microwave waves that fall into two categories: resonant (narrow band) [26,27] and non-resonant (broadband) [28,29,30]. Resonant techniques have grown in popularity among these, particularly for applications requiring high detection speed, precision, and sensitivity [31,32,33]. Because a greater range of frequencies can be covered to measure the microwave parameters of the materials and obtain more detailed information about the electromagnetic properties of the sample under test (SUT), increasing the number of resonances of sensor performance is one method to improve the accuracy of detecting electrical parameters of materials by microwave resonant sensors [33,34,35,36,37]. A band-stop filter with a defective ground structure (DGS) sensor is another technique that uses scattering parameters to monitor intravenous glucose levels, as reported in [38]. Moreover, it is less sensitive than previously reported to variations in blood sample glucose levels and penetration depth. However, there are several options presented that relate to checking blood glucose levels [39,40] using near-infrared and electrochemical sensors. In [41], a regular-shaped SRR sensor was used to monitor the glucose level. Moreover, Refs. [42,43] proposed a different pressure sensor, and Ref. [44] provided several creative microwave sensors, while Deshmukh and Ghongade devised a method for measuring glucose levels by estimating the glucose levels and computing the dielectric characteristics using a ring resonator [45]. The detection of glucose in the presence of animal tissues was addressed by the authors of [46] by using mm wave techniques. Furthermore, a variety of novel techniques for more precisely determining the glucose content in blood samples are described in [30,31,32,33,34,35,36,37,38,39,40]. The most popular non-invasive glucose monitoring technology is the antenna system because of its great sensitivity to even small fluctuations in blood glucose levels, ease of deployment, resistance to noise, temperature, and other disturbances, and deep penetration.

A variety of liquid phantoms, such as physiological solutions and pig blood, were assessed; the samples under analysis had glucose concentrations ranging from 150 mg/dL to 550 mg/dL. It was found that the solution caused a linear shift in frequency of 5 MHz, with no correlation between the readings and the samples’ varying volumes and temperatures [47]. In [48], metamaterials can also be used to identify glucose. In [48], the simulated result sensitivity for differences in a small range of glucose changes from 0 mg/dL to 200 mg/dL is very small, 0.017 dB/mg/dL at three operating bands.

**Figure 1:**
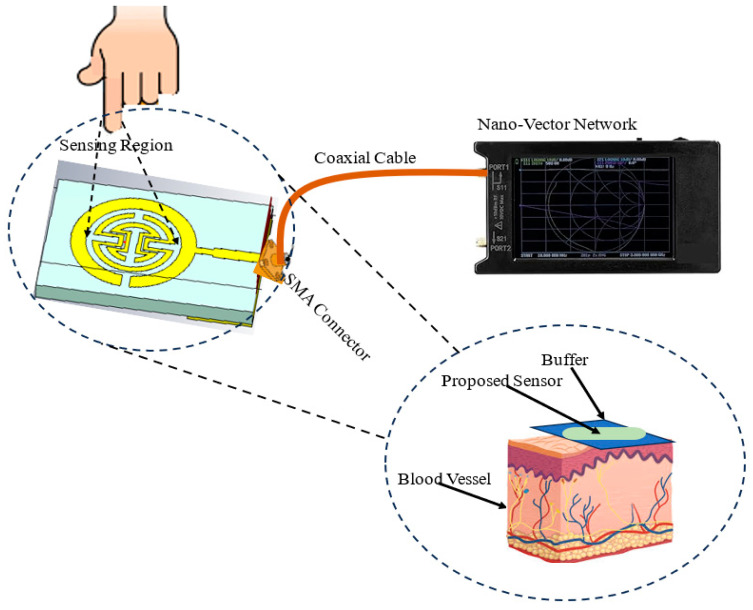
Diagram of the proposed sensor model.

As shown in Figure 1, this paper introduces a proposed biosensor system using a compact monopole antenna based on an irregularly shaped split-ring resonator (MSRRMA) for monitoring blood glucose levels. This design enables multiple resonant frequencies at 0.94, 1.5, 3, 4.6, 6.3, and 7.2 GHz, which is beneficial for monitoring blood glucose levels across a range of applications.

The proposed sensor allows for continuous monitoring of blood glucose levels without requiring invasive blood sampling. This represents a significant improvement over traditional glucose monitoring methods that require finger pricks or other invasive techniques. The incorporation of two SRR metamaterial cells to create specific notches at 1.7 GHz and 4.4 GHz is innovative. It enhances the antenna’s ability to respond to changes in blood glucose levels selectively. The proof of concept is carried out by using two technologies: containers having solutions with different glucose concentrations and the other one by using a human fingertip.

The proposed design could improve the sensitivity and specificity of the monitoring system by 24 MHz/mg/dL. Constructed on a commercial FR4 substrate with compact dimensions (35 × 50 × 1.6 mm^3^), the design is practical for real-world applications. The compactness of the antenna makes it suitable for wearable or portable devices. Moreover, the sensor is tested on real human fingers, measuring resonant frequency, magnitude, and phase changes. This method of validation provides strong evidence of the sensor’s effectiveness, enhancing the credibility of the research findings.

The paper is organized as follows: The material and methods used in the design process, parametric analysis, and microstrip antenna structure are all covered in Section 2. Results and comments are presented in Section 3, along with modeling of a study on frequency changes related to a finger phantom. Discussion and comments are presented in Section 4, along with modeling of a finger phantom-related investigation on frequency shifts for various finger phantom positions and glucose levels. Section 5 concludes the paper’s results.

## 2. Method and Material

### 2.1. Architectural Model Descriptions

**Figure 2:**
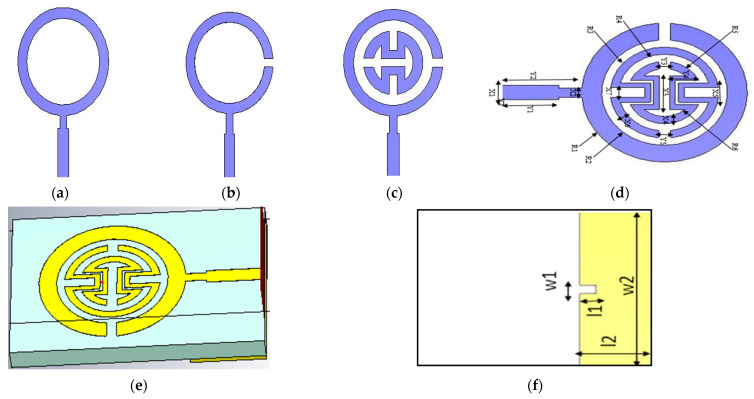
(**a**–**d**) Evolution of the proposed antenna design steps; (**e**) 3D-top side; (**f**) bottom side.

**Figure 3:**
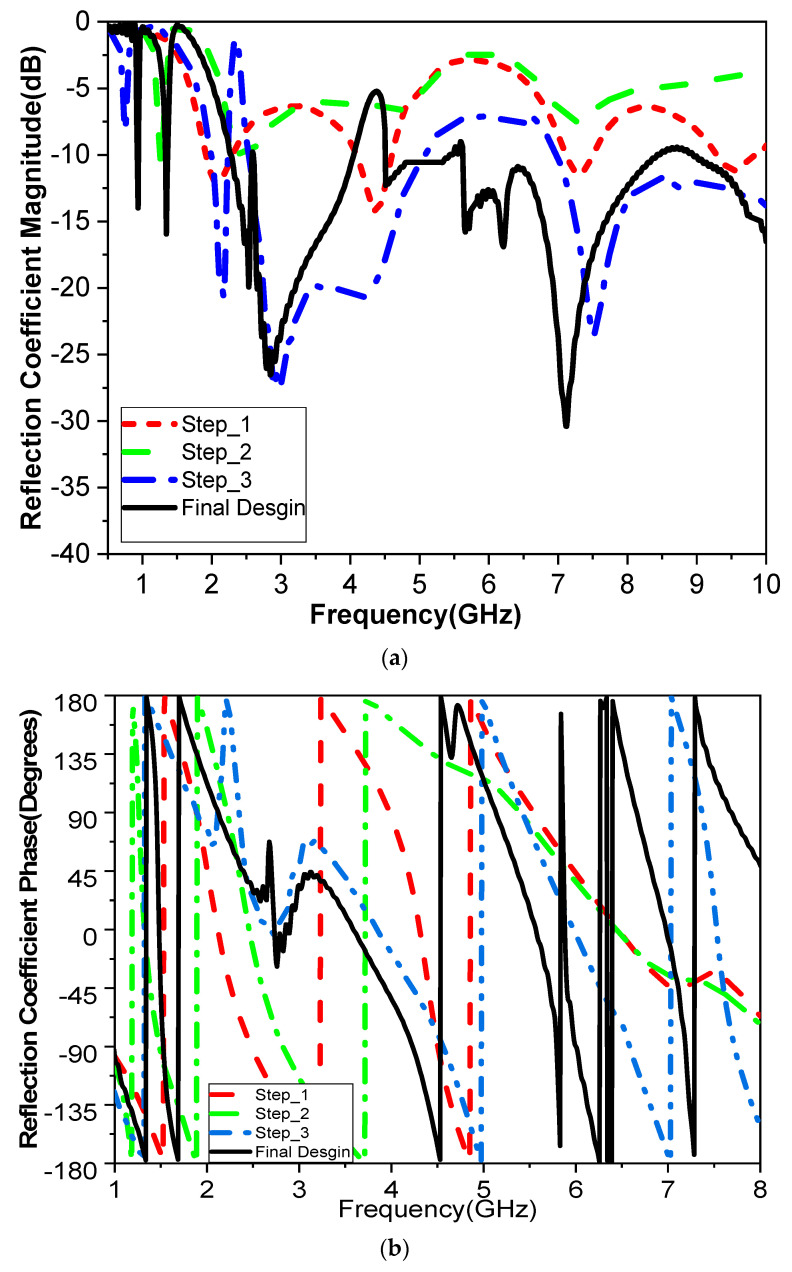
The simulated reflection coefficient results of the design steps are (**a**) magnitude and (**b**) phase.

The analysis was conducted using a CST electromagnetic field simulator, along with optimization of the proposed antenna sensor. The development of the antenna sensor progressed through various stages, as illustrated in Figure 2a–c, leading to the final design shown in Figure 2d. The antenna was constructed on a low-cost FR4 substrate with a thickness of 1.6 mm, a relative permittivity of 4.4, and a loss tangent of 0.02. The first step of design was a printed monopole antenna as a traditional elliptical sector shape, fed by a rectangular transmission line with step impedance to provide an input impedance of 50 Ω and improve matching at 2.1 GHz. The reflection coefficient response, magnitude and phase, at each design stage are shown in Figure 3. In the second step of the design, an air gap was etched with an elliptical sector shape to create a resonant frequency at 1.4 GHz, as shown in Figure 3, with a size reduction of 33%. This was implemented to increase the current path and reduce the resonant frequency from 2.1 GHz to 1.4 GHz. The |S_11_| response for this step is shown as the green dashed line in Figure 3. In the third step of design, an irregular curved shape was introduced as a first cell for the proposed split-ring resonator (SRR), as seen in Figure 2c. The |S_11_| response for this design stage is represented by the blue line in Figure 3. The resonant frequency was further reduced to 0.9 GHz, and a notch was created at 2.1 GHz, with a 57% reduction in size from the original shape. Another irregular curved SRR shape was then added for the final design, as shown in Figure 2d, forming a second cell of SRR, which produced a second notch at 4.4 GHz and reduced the first notch at 1.4 GHz, as shown in Figure 3. As depicted in Figure 2f, the antenna consists of a rectangular ground plane and an elliptical patch with major radius *W_2_* and minor radius *L*_1_. In the final phase of design, a U-shaped structure was etched onto the ground plane beneath the transmission line to improve impedance matching. This is represented by the black line in Figure 2e,f. The |S_11_| response shows an ultra-wideband (UWB) performance with two notches at 1.7 GHz and 4.4 GHz, achieving a sensor size reduction of about 57% from the original. The proposed sensor antenna offers better matching, reduced losses, and additional bands, maximizing measurement accuracy and detection scheme. The inclusion of SRRs enhances the antenna’s sensitivity by boosting coupling, which is essential for strong interactions with electromagnetic fields in the region of human finger positioning. The simulation parameters for the proposed sensor are listed in Table 1. Designing the proposed sensor with multiple resonant frequencies in glucose sensing allows for more precise, reliable, and selective detection of glucose. This highly suits complex biological systems with non-linear behavior and improves the overall performance of the sensing device. Moreover, it provides a broader dynamic range for detecting glucose levels across different concentrations. Different frequency bands may respond differently depending on glucose concentration, allowing the sensor to cover a wider range of glucose levels and improve its performance in real-world scenarios, such as in clinical diagnostics or portable devices.

Glucose exhibits distinct dielectric behaviors in the microwave frequency range. Specifically, the dielectric constant and loss factor of glucose vary significantly at certain frequencies due to glucose’s molecular polarization mechanisms (e.g., dipolar relaxation). The chosen frequencies at 0.94, 1.5, 3, 4.6, and 6.3 GHz correspond to regions where glucose’s dielectric response is most pronounced, as per recorded from measured properties for glucose solutions, shown in later, for improved sensitivity and accurate detection. The chosen frequencies are within the range commonly used in biomedical sensing applications, ensuring compatibility with existing technologies and minimizing signal attenuation in biological tissues. This makes the system practical for real-world, non-invasive glucose monitoring.

### 2.2. Glucose Characterization

Initially, the glucose samples with different concentrations were characterized using a DAK (dielectric assessment kit) to determine the different electric properties of the proposed sample concentrations. The DAK uses three open-ended coaxial dielectric probes, as described in [49]. This precision dielectric measuring device is intended to measure frequencies between 4 MHz and 67 GHz. The data are exported from the DAK and are imported into the 3D electromagnetic CST simulator to incorporate its effect with the collected response from the near-field sensor. There are two methodologies for identifying different solutions with various glucose concentration levels. In the frequency range of 0.1 to 100 GHz, the dielectric characteristics of glucose concentrations in water have been identified [50] and are presented later for the measured samples. Furthermore, measurements in the 30–100 GHz range were made of the glucose concentrations in a physiological salt solution, which is thought to be a blood imitator [51]. Additional results for varying glucose concentrations in water in the 0.5–20 GHz range are presented in this work. All the work referred to for characterizing glucose solutions with different concentrations shows distinct variations at the target bands. This allows for collecting a distinct response from the proposed sensor. Additionally, our group previously studied variations in the electrical properties of other blood composites, such lactate and urea [52]. Glucose, lactate, and urea each have distinct dielectric properties in the microwave frequency range [50,51].

#### 2.2.1. First Methodology

**Figure 4:**
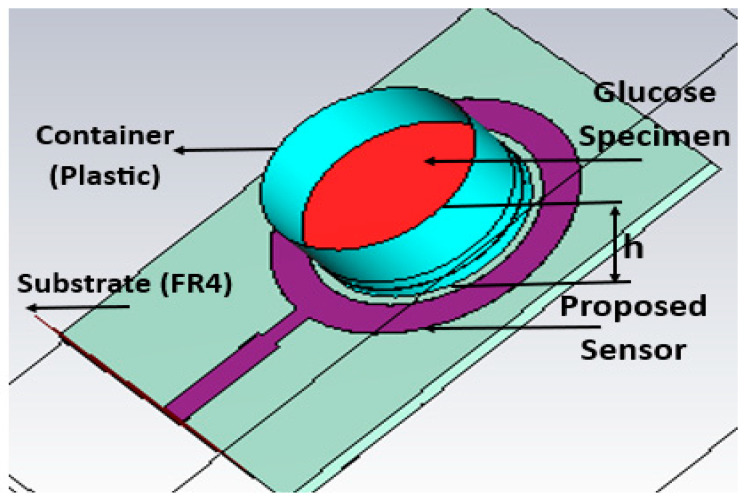
The simulation setup compares the different levels of glucose concentration.

The evaluation of various glucose concentrations was performed by simulating a plastic container with a dielectric constant of 2.2, as shown in Figure 4. The container, cylindrical in shape, was filled with equal amounts of water mixed with varying quantities of glucose to achieve specific concentration levels. It was placed over the proposed sensor, directly above the SRR region, where the electromagnetic field coupling is strong due to the intense interaction between the magnetic field and the container. Different glucose specimen levels were spectacled according to the glucose range of interest, from 0 to 350 mg/dL. This study used glucose–water solutions for baseline validation. The container was constructed of plastic and had an inner diameter of 12 mm, a thickness of 1 mm, and a height of 10 mm. Because the electric and magnetic fields were concentrated within the container, it was positioned over the SRR area to maximize interaction. The electrical properties, as permittivity’s real and imaginary values at different glucose concentrations at resonance frequency equal to 2.45 GHz, based on the Cole–Cole and Debye models, are shown in Table 2.

**Figure 5:**
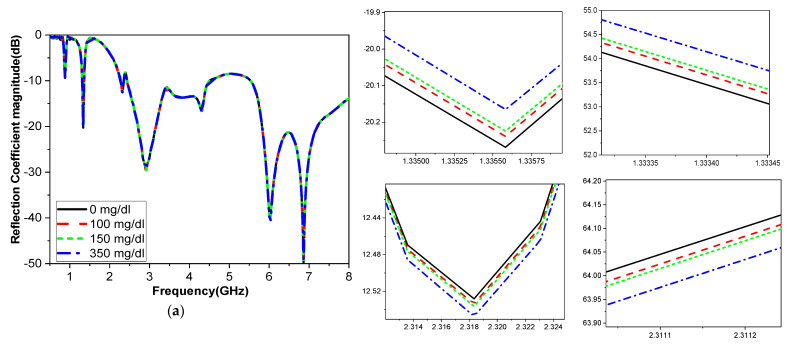

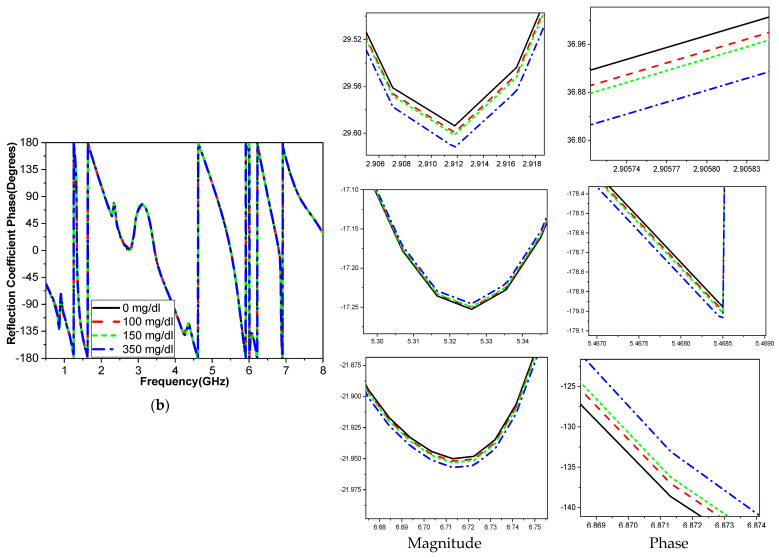
The phantom reflection coefficients for different glucose concentrations in simulations: (**a**) magnitude; (**b**) phase comparison.

The reflection coefficient response of the proposed sensor for different glucose concentrations is shown in Figure 5. The figure provides a zoomed-in view of the reflection coefficient behavior at various glucose concentrations. Different frequency bands, ranging from 1 GHz to 3 GHz, interact with glucose molecules in distinct ways, unlike the higher frequency bands above 4 GHz. By utilizing multiple bands, the sensor can take advantage of various resonant frequencies that correspond to glucose’s unique dielectric properties. This approach helps achieve more precise detection by capturing a broader range of interactions with glucose at different frequencies. Table 3 summarizes the results of the simulated frequency shifts and variations in the magnitude and phase of the reflection coefficient for the proposed sensor when a thin layer of glucose solution is applied in the container.

In the RF/microwave-based simulation system, the main component required is an antenna sensor, which is placed in proximity of the container full of glucose with different concentrations. Different parameters of the antenna sensor, such as reflection coefficient S_11_ (dB) and (degree), shift in resonant frequency, and electric/magnetic fields are then studied to correlate them with the glucose concentrations.

From the above table, the interaction between glucose concentration is not linear with frequencies; this is due to the interaction with electromagnetic waves in the radio frequencies, such as higher-order absorption or scattering. These non-linear behaviors as well as the molecular interactions, do not scale proportionally with increasing concentration for glucose-sensing problems.

#### 2.2.2. Second Methodology

The creation of an antenna sensor that can monitor changes in blood glucose level (BGL) from the fingertips is the main goal of this paper. To validate the operation with human measurements, a real human finger is mimicked by a phantom model created in a 3D electromagnetic software CST ver. 2018 environment. Dielectric materials with varied dielectric constants and conductivities are used to simulate the different layers of the finger, including skin, fat, muscle, blood, and bone. The thicknesses of the various layers of the finger phantom, together with their respective dielectric constants, are given in Table 4, based on the Cole–Cole and Debye parameter polynomials.

Variations in a person’s blood glucose level (BGL) can be detected by placing the finger on the proposed design sensor. Glucose levels can be sensed from two layers: blood and fat, with fat being preferable due to its higher glucose concentration. Differences in resonance shift (∆F), phase (degrees), and S_11_ magnitude are attributed to variations in weight or finger size of the body under test (BUT). To prevent finger pressure from affecting the results or causing a short circuit in the SRR, a 1 mm thick glass buffer is placed over the sensor. The buffer is constructed from glass bricks measuring approximately 5 × 2.5 cm^2^ and 1 mm in thickness. The effect of finger orientation on the surface of the proposed sensor is also examined to confirm that the sensor’s performance is only slightly influenced by the finger’s position, whether aligned with the SRR air gap or perpendicular to it.

**Figure 6:**
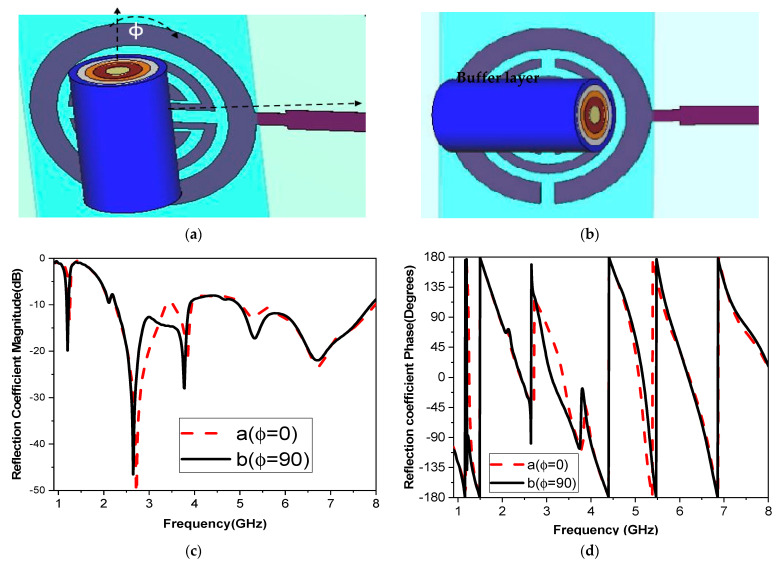
Sensor configuration of the finger simulation platform: (**a**) ɸ = 0°; (**b**) ɸ = 90°; (**c**) |S_11_| magnitude; (**d**) |S_11_| phase.

**Figure 7:**
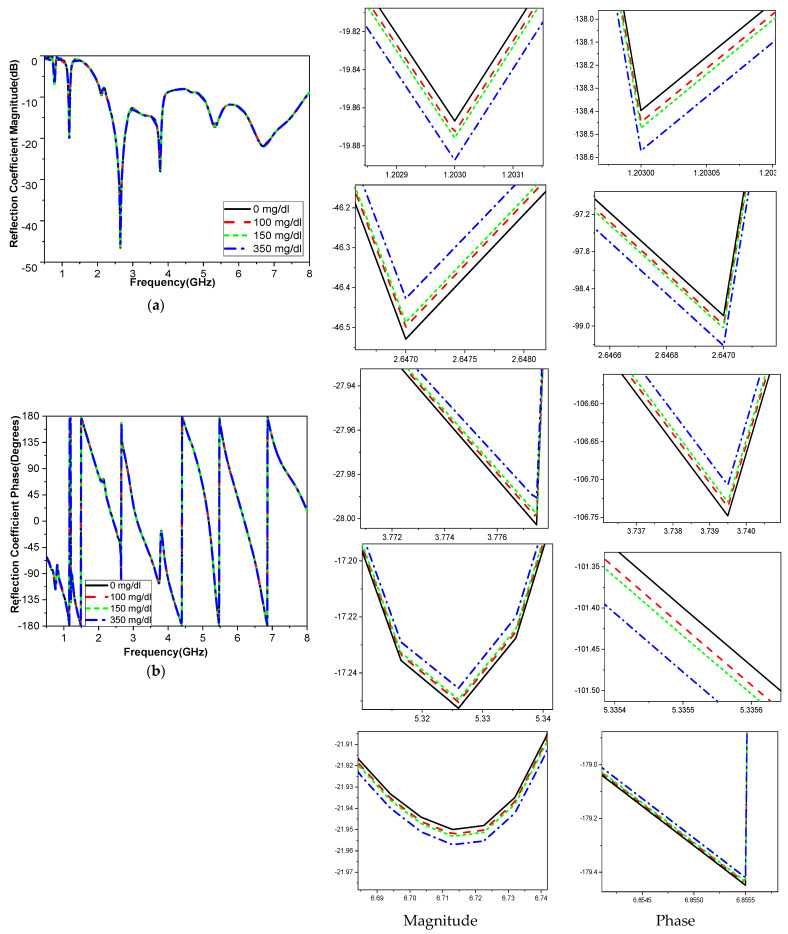
The antenna simulation’s S_11_ (**a**) magnitude and (**b**) phase of the platform finger’s varying concentration.

To analyze the impact of the human finger on the sensor, the finger is placed in two orientations using a buffer, as illustrated in Figure 6a,b. The buffer is necessary to mitigate the influence of PCB conductivity on the sensitivity of measurements with direct placement on biosensors. The finger is placed over the two-cell split-ring resonator (SRR) sensor in either alignment: parallel or perpendicular to the current path of the monopole feed line. Results from both configurations reveal similar performance in terms of reflection coefficient magnitude and phase, as shown in Figure 6c, d. These findings indicate that the orientation of the human finger has minimal impact and does not affect the accuracy of blood glucose readings. Figure 7 shows the simulated response of the sensor antenna with a human fingertip with different glucose levels: 100, 150, and 350 mg/dL, which are typical for daily life with diabetes.

The selectivity of our SRR-based sensor to glucose is achieved through many factors: The proposed SRR structure is designed to operate at frequencies that relatively acquires larger variations in the dielectric response of glucose. Moreover, the geometry and dimensions of the SRR structures are optimized to enhance sensitivity to the dielectric changes induced by glucose. The 1.7 GHz and 4.4 GHz notches correspond to resonant modes that are specifically tuned to detect the dielectric signature of glucose. Other metabolites such as lactate and urea may also interact with the SRR. However, in [52], recorded differences in dielectric properties for lactate and urea solutions with different concentrations in the microwave range are either negligible or do not align with the resonant modes of the SRR. This validates the sensor selectivity to glucose changes.

The finger phantom is placed above the radiating element of the proposed sensor, and changes in frequency, S_11_ magnitude, and phase are used to gauge fluctuations in the glucose level. Table 5 illustrates how the simulated operating frequency shifts when the fingertip is placed on the sensor surface with different BGL, as well as the reflection coefficient change in magnitude and phase. At various frequencies, the variation in relative permittivity brought on by glucose during the simulation is apparent within glucose values ranging from 0 to 350 mg/dL.

**Figure 8:**
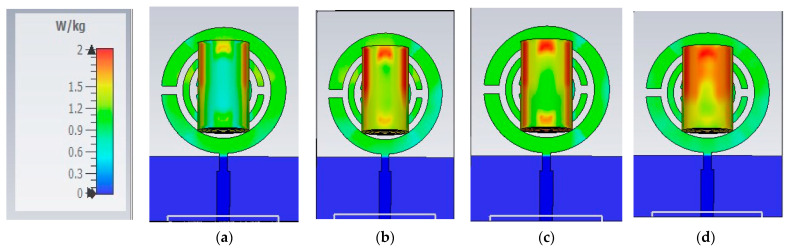
The proposed sensor SAR of the human finger is at (**a**) 1.2 GHz, (**b**) 2.64 GHz, (**c**) 3.77 GHz, and (**d**) 6.7 GHz.

The results in both Table 4 and Table 5 verify the sensitivity to glucose changes in some bands compared to others. Higher variations are recorded around the band in the range of 2–5 GHz. These findings will be further validated through experimental measurements using glucose solutions as well as fingertips from volunteers. Moreover, to guarantee that non-invasive blood glucose monitoring devices are safe, efficient, and comfortable for users and to comply with regulatory criteria, SAR calculations were conducted, as shown in Figure 8. Specific absorption rate (SAR) is strictly regulated by organizations such as the International Commission on Non-Ionizing Radiation Protection (ICNIRP) and the Federal Communications Commission (FCC) in the USA to ensure that electronic devices are safe for human use.

Compliance with these limits is essential for devices to receive the necessary approvals. The FCC and ICNIRP set SAR limits at 1.6 W/kg and 2.0 W/kg, averaging over 1 g and 10 g of tissue, respectively, with similar regulations in the European Union.

In this study, a finger phantom is placed above the radiating element of the proposed sensor to calculate the absorption power and ensure that the system is safe for human use, as illustrated in Figure 8. The maximum SAR value is approximately 2 W/kg at 6.7 GHz, while the average value over the operating band is around 2 W/kg.

## 3. Experimental Results

**Figure 9:**
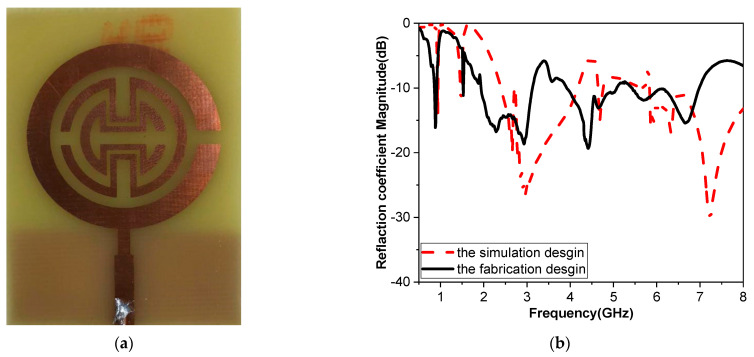
(**a**) Photo of the fabricated proposed sensor and (**b**) the comparison of |S_11_| simulated and measured values.

As shown in Figure 9, the multiband monopole antenna sensor was fabricated to validate the design results of the MSRRMA sensor. This sensor was fabricated in the Microstrip Department of the Electronics Research Institute by using photographic techniques to fabricate the printed circuit board (PCB). The MSRRMA sensor was measured on air, and then SAR was measured at different resonant frequencies and the two technologies were applied as plastic containers with different glucose concentrations and human fingertips.

### 3.1. Sensor Fabrication and Measurement in Air

Printed circuit boards (PCBs) were utilized in the fabrication of the proposed multiband monopole antenna sensor. To evaluate the design performance, a low-cost FR4 substrate was used in a precise photolithography technique, as shown in Figure 9. The sensor was fabricated in the Microstrip section of the Electronics Research Institute. The reflection coefficient of the proposed sensor was measured in the air using the Rohde & Schwarz ZVA 67 Vector Network Analyzer, Munich, Germany. From Figure 9, there is good agreement between the simulated and measured results; however, some variations are inevitable. Experimental measurements could be affected by various sources of error, such as instrument precision, calibration issues, or human error during sample preparation or data collection and the effect of soldering. Understanding and accounting for these differences can help refine the design and testing processes. The discrepancies may be due to the idealized conditions in simulations compared to real-world factors. Enhancements in soldering techniques, accurate material characterization, manufacturing processes, and regular calibration can significantly reduce the gap between simulated and measured outcomes.

### 3.2. SAR Measurement

**Figure 10:**
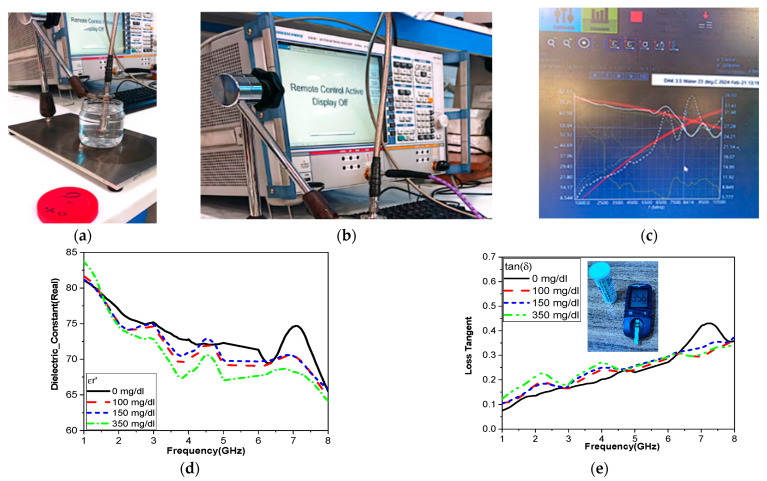
(**a**–**c**) Measurement by using the DAK; (**d**) actual dielectric constant; (**e**) tangent loss.

SAR measurements are essential for ensuring the security of wireless devices since they quantify the exposure to electromagnetic fields. Manufacturers may verify that their devices are safe and reliable in real-world environments by following standard operating procedures and using calibrated equipment, as illustrated in Figure 10, at the Electronics Research Institute, Central Laboratory. The proposed sensor’s observed SAR values at 1.5, 3 GHz, and 5.2 GHz are displayed in Table 6. According to Table 6, the SAR values obtained for the proposed sensor at different power levels fall within the accepted safe limits as stated by the IEEE standard, which is 1.6 W/kg. As demonstrated, the SAR is measured at various resonance frequencies and input powers. The SAR is measured at different resonant frequencies and input power levels, as shown in Table 6.

### 3.3. Result of the Glucose Characterization

The glucose concentration was prepared by dissolving the prescribed amounts of glucose in distilled water to yield concentrations of 0 mg/dL, 100 mg/dL, 200 mg/dL, and 350 mg/dL. Next, we entered the various concentrations into a DAK device to measure the properties of the glucose concentration, such as permittivity real value and loss tangent over the operating band, as shown in Figure 10.

First, an inverse relationship between the magnitude of the dielectric constant and the value of glucose level were recorded. When the value of the glucose concentration level was increased, the real value of the dielectric constant decreased, as shown in Figure 10. Additionally, the variation in the permittivity within the operating band from 3 to 4 GHz and from 5 to 6 GHz was larger than other measured operating bands. Second, on the contrary, the imaginary part of the glucose concentration level increased with the value of the glucose level.

The relationship between the S_11_ parameter and glucose concentration can be explained by changes in the effective relative dielectric constant (ε_ref_) of the glucose solution. As glucose concentration increases, ε_ref_ decreases due to the lower permittivity of glucose compared to water. This reduction in ε_ref_ alters the propagation constant (β) and, consequently, the reflection behavior of the sensor, as indicated by Equation (1).

(1) $$\beta =\frac{w}{C}\sqrt{\varepsilon_{ref}}$$

where w is the operating frequency, C is the speed of light, and ε_ref_ is the relative effective dielectric constant.

As εref decreases, the electromagnetic wave propagates with less attenuation, increasing the reflection coefficient *S*_11_ loss. The reflection coefficient (*S*_11_) is calculated as Equation (2).

(2) $$S_{11}=\frac{V^{-}}{V^{+}}=\frac{V_{ref}}{V_{inc}}$$

where Vref and Vi*n*c are the reflected and incident signal voltages. When εref decreases, Vref decreases slightly, resulting in a lower *S*_11_ loss. This behavior is reflected in the frequency response of the sensor. The perturbation in the resonant frequency ∆f due to a change in the dielectric constant ∆*εr* is given by Equation (3).

(3) $$∆f=\frac{f_{r}}{2}\sqrt{\frac{{∆\varepsilon }_{ref}}{\varepsilon_{ref}}}$$

where f*_r_* represents the resonant frequency.

### 3.4. Measurements of Proposed Sensor with Glucose

This part contains comparable methodologies to the measurement of glucose levels using the plastic container and human finger. The container was employed at specific concentration levels during characterization. A glucometer was used to measure this concentration.

#### 3.4.1. Measurements with Glucose Solutions with Container

**Figure 11:**
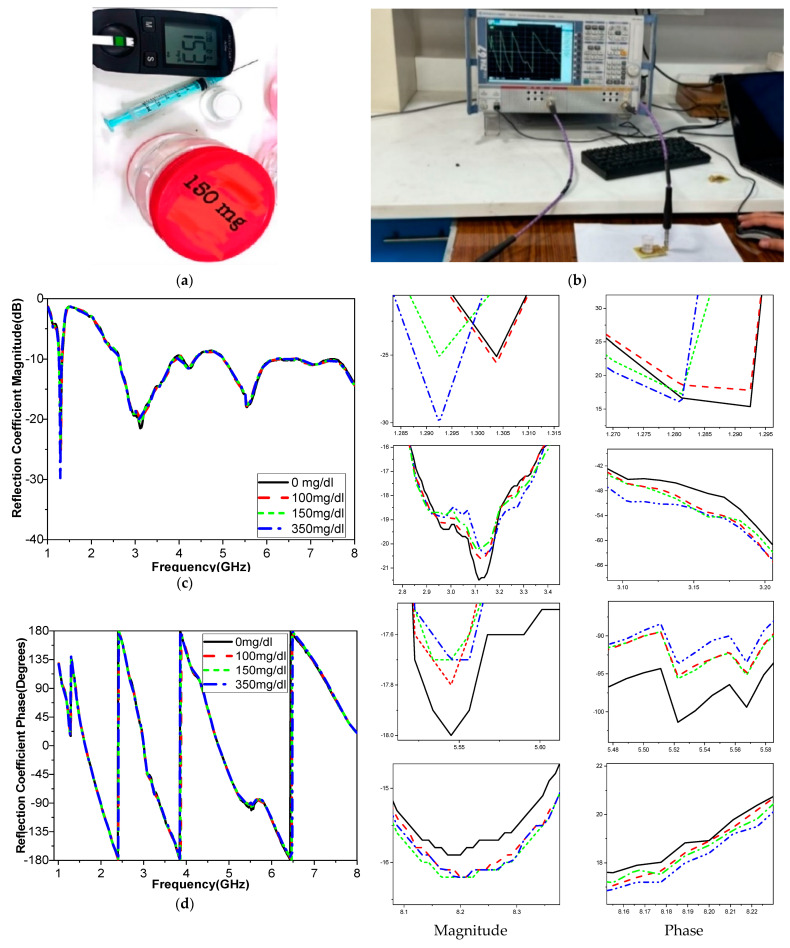
(**a**) Proposed antenna sensor loaded with a container filled with glucose content. (**b**) Photo of the measurement setup. |S_11_| at different glucose concentrations: (**c**) magnitude and (**d**) |S_11_| phase.

The recommended reflection sensor’s performance was measured and reported. A dielectric container was placed on the proposed fabricated sensor’s top to release the liquid into the sensing area. A plastic container with an ε_r_ = 2.2 component was placed symmetrically over the sensor region using a Rohde and Schwarz VNA, as seen in Figure 11a. The container’s base measured 12.6 × 2 × 1 mm^3^. Using a pipette, various concentrations of glucose (0, 100, 150, and 350 mg/dL) were administered; the same amount is used for each concentration. The results of different concentrations of the transmission and reflection coefficients are shown in Figure 11. By analyzing the shifts in the frequency, magnitude, and phase of the S-parameter, as shown in Figure 11, the difference between the glucose concentrations in the container was determined by measuring differences in the S-parameters.

The highest shift in frequency, magnitude, and phase were recorded for the 350 Mg/dL concentration. The changes in S_11_ across the spectrum are highly non-linear, primarily due to the interaction between the sensor’s electromagnetic fields and the sample significant variation across the frequency range, leading to a non-uniform response. Further complicating the response patterns, noise across the spectrum introduces additional variability.

#### 3.4.2. Measurements with Human Finger

In the second case, fingertip measurements are validated by monitoring glucose levels for four volunteers. Finger measurements might be a useful technique for determining how various blood levels interact with one another. Human fingers were used. Therefore, approval from an Ethical Perspective NILES-EC-CU 24/1/2, issued on August 2024, was obtained. The measuring process requires participants (healthy and diabetic participants) to fast for six hours before the test. After checking their glucose levels, the participants are re-tested two hours after eating, as a second round of testing. Glucose levels of participants are expected to reach higher values compared to prior status while fasting. Before the test began, the participants washed their hands to remove any contaminants, and they were given around fifteen minutes to relax until their bodies stabilized.

**Figure 12:**
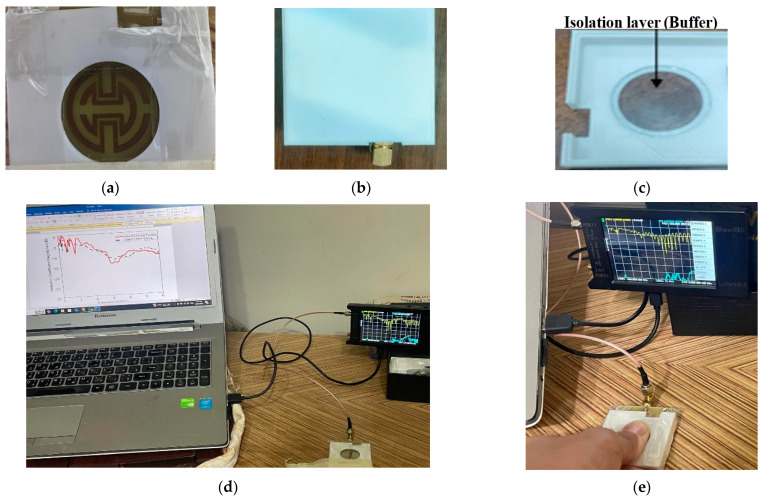
The housing of the sensor: (**a**) front view; (**b**) back view; (**c**) detailed view of the measurement setup; (**d**) without the finger; (**e**) with the finger.

All measurements were conducted in a controlled environment to minimize external factors such as movement and temperature variations that could impact the readings. Participants were asked to remain seated and avoid finger movement during the measurements. To ensure consistent contact between the fingertip and the sensor, the sensor housing was designed with a fixed position guide that helps maintain a uniform fingertip placement, as shown in Figure 12. To mitigate this, the sensor housing includes a small hole with an isolation layer that ensures consistent contact pressure, as shown in Figure 12a–c. This guide reduces the variations caused by different fingertip placements and finger pressure.

**Figure 13:**
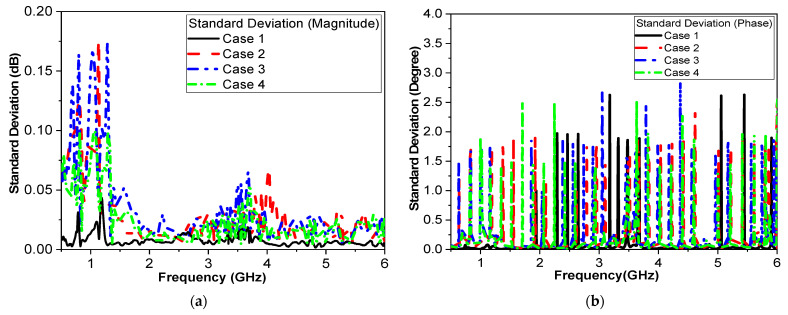
Standard deviation across 10 readings for each of the four participants. Reflection coefficient (**a**) magnitude and (**b**) phase.

Our proposed approach involves designing and fabricating a custom packaging case using 3D printing technology, which would afford us the necessary flexibility in design to optimize sensor performance. This case will enclose the sensor and incorporate a plastic or insulating layer to mitigate the influence of human tissues on sensor readings. The S_11_ shifts are correlated with blood glucose measurements obtained through conventional glucometer readings. The results showed a strong correlation between the changes in the S_11_ profile and the BGL levels measured by the glucometer. This supports the claim that the shifts are primarily due to changes in BGL rather than other external factors. Data from four volunteers in the study are shown in Table 7, with each subject undergoing 10 trials. This allowed us to collect a substantial dataset to evaluate the sensor’s repeatability and reliability across different individuals and conditions. To assess repeatability error, the standard deviation of the S_11_ magnitude and phase measurements across the 10 trials for each subject was calculated. The results in Figure 13 show that the variability was within an acceptable range, demonstrating the sensor’s repeatability. Moreover, the data show that the S_11_ phase exhibited a lower standard deviation than the S_11_ magnitude, indicating that the S_11_ phase readings have higher repeatability and are less susceptible to measurement variability, thus showing greater robustness.

**Figure 14:**
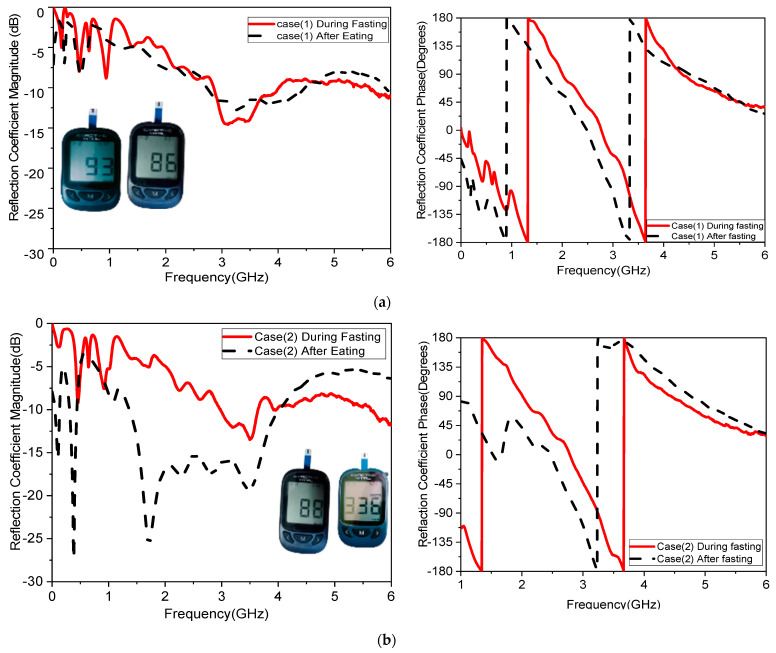

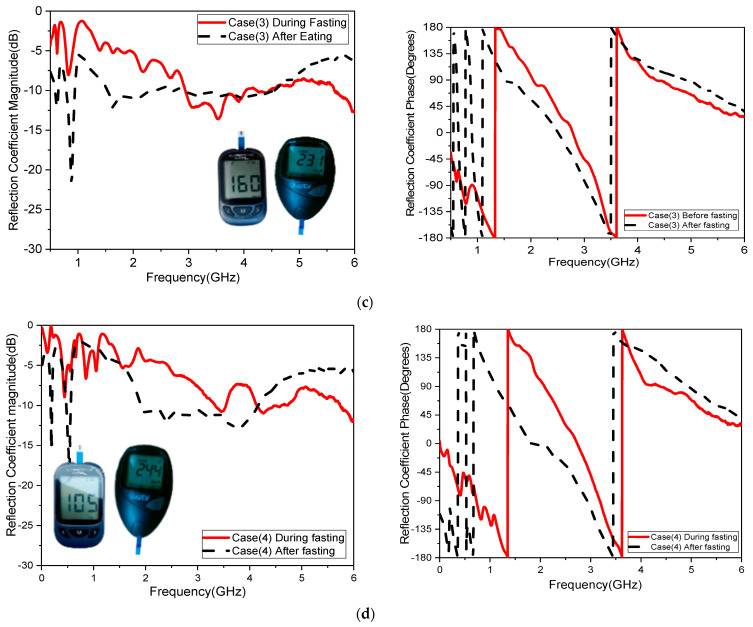
Measurements using the proposed sensor: |S_11_| magnitude and phase during and after fasting for (**a**) case 1, (**b**) case 2, (**c**) case 3, and (**d**) case 4.

To ensure the stability of the sensor and mitigate the effects of fingertip movement during the trials, the sensor housing was designed with a fixed-position housing to secure the fingertip consistently during each measurement. This guide helped maintain uniform positioning and pressure, reducing the likelihood of movement artifacts. Different case studies were used; two normal blood glucose levels (one male and one female) and two diabetic levels (one male and one female). The glucose concentration was measured using the Nano VNA during fasting and after eating for a total of four cases. The concentration of glucose in the blood was initially measured using a glucometer as well as the Nano VNA after calibration, as shown in Figure 14. The magnitude and phase of the S-parameters were recorded in the four cases and are presented in Figure 14.

The glucose level in the blood was first measured with a glucometer and is presented in Table 7. As shown in Figure 14, after measuring the four distinct cases and comparing the results during and after fasting, the difference between the measurements made after and before fasting is determined by examining the shift and frequency changes. The next section discusses the differences in the results.

## 4. Discussion

The frequency band is varied and tested throughout a range to determine how variations in glucose concentration affect the reflection coefficient, which was utilized to compute the sensor’s sensitivity. When blood is added, the interaction between the blood glucose samples and the fields modifies the resonant frequency for the relative permittivity εr and the sensitivity of the sensor. The variation in the glucose concentration changed according to the resonant frequency of operation, as shown in Figure 14. The operating bands 3.1 GHz and 5.95 GHz are more sensitive frequency bands to the variation in the blood glucose concentration. This corroborates with the results shown in Figure 10. Equation (2) is used to analyze the sensitivity; it shows that any change in the sensor’s resonant frequency (Δf) or magnitude (ΔS_11_) depends on a change in the glucose concentration (ΔC). One may extract Equations (4)–(6), as in [5].

(4) $$S_{fr}=\frac{∆F}{∆C}\;\;\;\;MHz/mg/dL$$

(5) $$S_{dB}=\frac{∆S}{∆C}\;\;\;\;dB/mg/dL$$

(6) $$S_{Ph}=\frac{∆\phi }{∆C}\;\;\;\;degree/{mgdL}^{-1}$$

The sensitivity of the measurement by using a container is around 0.015 MHz/mg/dL, 0.003 dB/mg/dL, and 0.003 degree/mg/dL at resonant 3.1 GHz 4.624. When at resonant frequency 5.9 GHz, the sensitivity is 0.5 MHz/mg/dL, 0.005 dB/mg/dL, and 0.05 degree/mg/dL. The measurement sensitivity using a human finger is around 0.299 dB/mg/dL for case 1 and 1.859 dB/mg/dL for case 2, and the sensitivity is around 0.05951 degree/mg/dL for case 1 and 0.510473 degree/mg/dl for case 2.

Moreover, the sensitivity is around 0.2427 MHz/mg/dL for case 1 and 24.624 MHz/mg/dl for case 2, while the value of sensitivity is around 0.299 dB/mg/dL for case 1 and 1.859 dB/mg/dL for case 2, and the sensitivity is around 0.05951 degree/mg/dL for case 1 and 0.510473 degree/mg/dL for case 2. The resonant frequency at 172.37 MHz decreases with increasing glucose concentrations between 3.280 and 3.1080 GHz. The frequency moves somewhat downward at lower levels and drastically lowers at higher quantities, as shown in Table 8. This alteration causes relative permittivity to drop and sensor sensitivity to increase with glucose concentrations. Our measurement system is designed to minimize the influence of external factors, including perspiration. Measurements are conducted in a controlled environment where factors such as humidity and temperature are regulated to reduce the likelihood of excessive perspiration. Participants are instructed to clean and dry their fingers before measurements to ensure consistent conditions. This step helps remove any existing moisture or contaminants that could affect the results. The sensor interface buffer is designed to maintain consistent contact with the skin, even in the presence of minor moisture. The materials and design of the sensor ensure that perspiration does not significantly alter the electrical or dielectric properties being measured.

One of the objectives in the proposed study was to record sensor response while testing with humans. Four volunteers were involved in testing as a first round of measurements. These measurements provide a proof-of-concept for the sensor’s sensitivity to glucose variations within a human finger. While the small sample size limits the generalizability of the findings, consistent and significant frequency shifts were observed in the volunteers. Figure 14 and Table 8 provide strong preliminary evidence of the sensor’s functionality. In future studies, the influence of finger size, skin thickness, and other physiological factors on the sensor’s performance is planned. Having ethical approval, more measurements could be collected from larger number of volunteers.

Table 9 compares the performance of the proposed sensor relative to that reported in the literature, in terms of sensitivity and size. The vision for the proposed sensor is to replace the traditional glucometer without the invasive Pickering operation at each measurement. The proposed sensor fits better into the portable category than the wearable one. In terms of cost, the proposed sensor costs 10 USD. With potential additional complementary circuit and display, 10 USD more, for an overall cost of 20 USD, compared to a 70 USD traditional glucometer.

Table 9 presents an evaluation of the data collected from the newly developed microwave antenna sensor compared with different configurations that have been previously published. To compare the results of this research with those of previous studies, Table 9 shows that the proposed sensor is more compact and has better sensitivity than the others. The proposed design, MSRRMA, demonstrates several advantages over other configurations in terms of practical applicability, sensitivity, safety, and versatility. The MSRRMA has a size of 35 × 50 mm^2^, which is relatively compact compared to most alternatives. For instance, the DR sensor and Microstrip Patch Antenna have larger areas ([5] 40 × 40 × 7 mm^2^ and [59] 43 × 43.6 mm^2^, respectively), making them bulkier and less practical for wearable or portable applications. The MSRRMA’s size allows for better integration into modern mobile devices. Unlike several configurations, such as the DR sensor [5], coplanar waveguide [56], and cylindrical CPW [57], the MSRRMA has been tested with humans. This practical validation ensures its reliability in real-world conditions and gives it an advantage over designs that are tested only in simulated or non-biological environments. Moreover, the proposed sensor design effectively measures glucose concentrations in the range of 0–350 mg/dL, which covers a substantial spectrum of clinically relevant glucose levels. While some designs like the coplanar waveguide [56] support a wider range (0–4800 mg/dL), the MSRRMA’s range is sufficient for monitoring hyperglycemia and hypoglycemia. The proposed sensor operates across a wide resonant frequency range of 0.5–7 GHz, enabling greater flexibility and adaptability. This range outperforms configurations like that reported in [54], which is limited to 1.45–1.55 GHz and [53] fixed at 6.1 GHz. Furthermore, the proposed sensor has a specific absorption rate (SAR) of 0.687 W/Kg at dBm for 10 g, which aligns with safety standards for electromagnetic exposure. The configuration reported in [58] has an SAR of 0.05 W/Kg, and the author did not mention at which power level.

The MSRRMA slightly exceeds but still complies with acceptable limits while offering better functionality at less than 2 W/Kg, and the others did not measure or calculate the SAR; designs without SAR evaluation cannot guarantee the same safety level. Compared to other sensors, the MSRRMA combines essential features such as compactness, human trial validation, broad frequency range, and safety. While some designs may excel in specific metrics (e.g., [5] in sensitivity), they often lack feasibility for wearable or practical medical applications. Finally, the MSRRMA exhibits a sensitivity of 24.6 MHz/mg/dL, showcasing its ability to detect minor glucose concentration variations. While the work in [5] shows a higher sensitivity (36.86 MHz/mg/dL), the MSRRMA strikes a balance between high sensitivity and other critical factors like compactness, safety, and practical implementation.

The proposed multiband sensor targets specific frequency ranges where glucose exhibits distinctive dielectric responses. This selective sensitivity to glucose-related changes, as opposed to other biological variations, improves the sensor’s specificity and reduces interference from other blood components or physiological factors. Existing designs may lack this fine-tuned specificity, leading to higher susceptibility to noise or non-glucose-related variations in the measurements. Additionally, this approach involves designing and fabricating a custom packaging case using 3D printing technology, providing the flexibility needed to optimize sensor performance.

Selecting lower operating frequencies from 1 to 5 GHz also offers significant practical advantages over high frequency. Sensors within this frequency range are easier to integrate with existing electronic devices and systems. In addition, sensitivity should increase with frequency due to the enhanced electromagnetic interaction at higher frequencies. Otherwise operating in a low-frequency range, the proposed sensor achieves superior sensitivity compared to high-frequency designs.

In future work, evaluating the impact of sweat, movement, temperature, blood type, and finger pressure on measurement accuracy may be necessary to incorporate compensation techniques. The sensor may require calibration for different finger sizes or the ability to adapt to a wide range of tissue volumes. Advanced signal processing techniques or machine learning algorithms could be employed to account for finger size variations and enhance measurement accuracy across different users. More work will be conducted on the selectivity of glucose compared to other composites and electrolytes mixed in blood, and designing additional circuits to complement the operation of the proposed system.

## 5. Conclusions

In this paper, a biosensor was designed based on MSRRMA for non-invasive glucose level measurement via the fingertip, operating at 0.94, 1.5, 3, and 4.6 GHz, with a sensor size reduction of approximately 57.5%. To simulate realistic conditions, a finger phantom model comprising five layers was developed in CST EM simulations. Each layer had variable dielectric properties, with the blood’s dielectric constant varying according to glucose concentration. The phantom was positioned at different locations along the antenna’s radiating element, with glucose concentrations ranging from 0 to 350 mg/dL. A sensitivity of up to 24 MHz/mg/dL was achieved when the phantom was placed directly above the antenna. Additionally, the specific absorption rate remained below the safety limit of 1.6 W/kg at a maximum power output of 20 dBm. The developed prototype was tested on volunteers, including both healthy and diabetic individuals, under fasting conditions and after eating. Significant variations in glucose levels were observed between the simulations and real-world measurements, demonstrating the accuracy of the phantom model in mimicking a human finger. With its compact size, high sensitivity, and cost-effectiveness, the proposed antenna-based sensor proves to be a promising solution for non-invasive blood glucose monitoring.

## Figures and Tables

**Table 1:** Dimensions of the proposed antenna sensor.

**Parameters**	**W1**	**W2**	**L1**	**L2**	**X1**	**X2**	**X3**	**X4**	**X5**	**X6**	**X7**	**Y1**
**Dimension (mm)**	2	35	5.1	16.6	3	1.92	4	1	5	1	7	11.5
**Parameters**	**Y2**	**Y3**	**Y4**	**Y5**	**Y6**	**R1**	**R2**	**R3**	**R4**	**R5**	**R6**	**t**
**Dimension (mm)**	17	2	7	2	5.1	14.5	11	9.5	8	6.5	5	0.035

**Table 2:** The electrical properties of glucose levels at 2.45 GHz.

Glucose Concentration (mg/dL)	$\varepsilon_{r}$	σ (S/m)
0	69.8470	3.4518
100	69.8203	3.4405
150	69.8070	3.4350
350	69.7550	3.4130

**Table 3:** Effect of the glucose solution in the container with the MSRRMA sensor.

Glucose Concentration	100 mg/dL	150 mg/dL	350 mg/dL
$∆f\left(KHz\right)$ **in 1st Resonance**	0.35	0.42	0.35
$∆f\left(KHz\right)$ **in 2nd Resonance**	1.83	1.83	2.5
$∆f\left(KHz\right)$ **in 3rd Resonance**	43.1 kHz	50.29	43.1 kHz
$∆f\left(KHz\right)$ **in 4th Resonance**	1.7	1.7	1.7
$∆f\left(KHz\right)$ **in 5th Resonance**	2	2.5	3
**Average** $\;∆S11\left(dB\right)$	6.188$\;\times \;10^{-3}$	9.0252$\;\times \;10^{-3}$	0.02046
**Average** ∆∅ **(Degree)**	0.7536	1.4897	2.6185

**Table 4:** Electrical properties of human finger layers at frequency 2.45 GHz.

**Phantom Parameters**	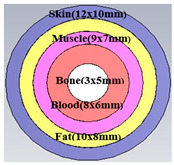	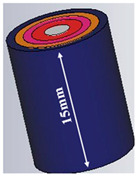	**Dimension** **(Radius × Thickness)**	$\varepsilon_{r}$	σ	**tan δ**
**Skin**			3 × 5	34.9	3.9	0.3336
**Fat**			6 × 8	4.9	0.3	0.1871
**Muscle**			7 × 9	48.1	0.3	0.3263
**Blood**			9 × 10	52	6.9	0.3933
**Bone**			13 × 10	9.54	1.2	0.3788

**Table 5:** Effect of the glucose solution in the figure tip with the MSRRMA sensor.

Glucose Concentration	100 mg/dL	150 mg/dL	350 mg/dL
$∆f\left(KHz\right)$ **in 1st Resonance**	0.33	0.27	0
$∆f\left(KHz\right)$ **in 2nd Resonance**	3.03	0.68	1.34
$∆f\left(KHz\right)$ **in 3rd Resonance**	15.2	2.17	28.24
$∆f\left(KHz\right)$ **in 4th Resonance**	5	5	5.5
$∆f\left(KHz\right)$ **in 5th Resonance**	5	8	9
**Average** $\;∆S11\left(dB\right)$	6.0319 × $10^{-3}$	8.8216 × $10^{-3}$	0.025084
**Average** ∆∅ **(Degree)**	1.039 × $10^{-3}$	1.175 × $10^{-3}$	2.651 × $10^{-3}$

**Table 6:** Comparison of the measured SAR levels for the proposed sensor.

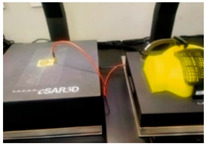		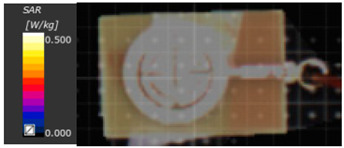					
	**(Fre). (GHz)**	**1.5**		**3**		**5.2**	
**P (dBm)…**							
		1 g	10 g	1 g	10 g	1 g	10 g
**0**		0.009	0.1	0.01	0.15	0.131	0.032
**5**		0.04	0.17	0.087	0.42	0.181	0.045
**10**		0.131	0.057	0.273	0.131	0.231	0.068
**15**		0.413	0.179	0.851	0.410	0.738	0.217
**20**		1.32	0.572	1.6	1.31	1.34	0.687

**Table 7:** Different case studies during and after eating.

Case #	With/Without Diabetes	Gender	Age	Glucose Level During Fasting	Glucose Level After Eating
**1**	Without	Female	48	86	93
**2**	Without	Male	65	88	336
**3**	With	Male	50	160	231
**4**	With	Female	45	105	244

**Table 8:** Outcome of the four cases when using the equations given in the text.

	(Case 1)	(Case 2)	(Case 3)	(Case 4)
**S_fr_ (** $MHz/mg{dL}^{-1}$ **)**	0.2427	4.624	3.4199	1.5387
**S_dB_ (** $dB/{mgdL}^{-1}$ **)**	0.299	1.859	0.012344	0.128014
**S_ph_ (** $degree/{mgdL}^{-1}$ **)**	0.05951	0.510473	$0.895\times 10^{-3}$	$3.887\times 10^{-3}$

**Table 9:** Glucose-level evaluations for different configurations of microwave sensors.

Measurement Technique	Ref	Area (mm^2^)	Human Trial	Concentration (mg/dL)	Resonant Freq. (GHz)	SAR W/Kg	S (MHz per mg/dL)
DR sensor	[5]	40 × 40 × 7	No	50–2000	5.89 to 6.09	No	36.86
Narrow-Band Microstrip Antenna	[53]	30 × 30	Yes	0–500	6.1	No	2.4
Circular Slot Antenna	[54]	25 × 25	Yes	0–1.81	1.45–1.55	0.05	0.5–0.8 dB.
Quasi Antenna Arrays	[55]	NM	Yes	79–408	0.5–4	No	NM
Coplanar waveguide	[56]		No	0–4800	2.4	No	0.004
Cylindrical CPW	[57]	22 × 12	No	60–240	2.4	No	0.430
CSRR resonator	[58]	59 × 20	NM	70–150	1–6	No	67–11
Microstrip Patch Antenna	[59]	43 × 43.6	No	0–400	2.4	No	25
Single-port Sensor	[60]	55 × 30	No.	100–1000	4.8	No	14
MSRRMA	Proposed	35 × 50	Yes	0–350	0.5–7	0.687	24.6

NM: not mentioned.

## Data Availability

The data that may be required to reproduce the findings of this study are available upon reasonable request from the corresponding author.
